# Gender Difference in the Association of Early- vs. Late-Onset Type 2 Diabetes with Non-Fatal Microvascular Disease in China: A Cross-sectional Study

**DOI:** 10.3389/fendo.2018.00015

**Published:** 2018-01-31

**Authors:** Xiaoxu Huo, Junqing Zhang, Xiaohui Guo, Juming Lu, Jing Li, Wei Zhao, Linong Ji, Xilin Yang

**Affiliations:** ^1^Department of Epidemiology and Biostatistics, School of Public Health, Tianjin Medical University, Tianjin, China; ^2^Department of Endocrinology, Peking University First Hospital, Beijing, China; ^3^Department of Endocrinology, Chinese PLA General Hospital, Beijing, China; ^4^Department of Endocrinology, Peking University People’s Hospital, Beijing, China

**Keywords:** early-onset type 2 diabetes mellitus, gender difference, diabetic retinopathy, diabetic nephropathy, Chinese

## Abstract

**Background:**

This study aimed to test whether early-onset (defined as <40 years of age) type 2 diabetes mellitus (T2DM) imparted different risks of microvascular disease to Chinese men and women.

**Methods:**

222,537 Chinese patients with T2DM were recruited in 630 hospitals from 106 cities in 30 provinces of China in 2012 using a cross-sectional design. Logistic regression analysis was performed to obtain odds ratios (ORs) of male vs. female for diabetic retinopathy (DR) and diabetic nephropathy (DN). Additive interaction was used to test whether male gender and early-onset T2DM had interactive effects for DR and DN.

**Results:**

More men than women with T2DM had DN (4.5 vs. 3.0%, *P* < 0.0001), DR (5.3 vs. 5.1%, *P* < 0.0001), and microvascular disease (either DN or DR) (8.4 vs. 7.1%, *P* < 0.0001). After adjustment for age and levels of hospitals, the effect sizes of early-onset T2DM for microvascular disease were higher in men than in women, with a 2.67 [95% confidence intervals (CI): 2.51–2.85] fold risk in men and a 2.53 (95% CI: 2.35–2.72) fold risk in women. The risk effect sizes were greatly attenuated by further adjusting for diabetes durations and other traditional risk factors, with a 1.28 (95% CI: 1.19–1.37) fold risk in men and a 1.07 (95% CI: 0.99–1.16) fold risk in women. After adjustment for diabetes durations and other traditional risk factors, using women with late-onset T2DM as the reference, co-presence of early-onset and male gender significantly enhanced the ORs of either early-onset alone (1.10, 95% CI: 1.03–1.19) or male gender alone (0.96, 95% CI: 0.93–0.99) to 1.32 (95% CI: 1.24–1.41), with significant additive interaction. Kaplan–Meier analysis showed that in early-onset T2DM, DN developed 5 years earlier in men than in women.

**Conclusion:**

Early-onset T2DM increased more risk of microvascular complications in Chinese men than in women, most of increased risks being attributable to longer diabetes durations.

## Introduction

The prevalence of type 2 diabetes mellitus (T2DM) is increasing worldwide, but its onset age is decreasing (1, 2). In mainland China, the prevalence of early-onset diabetes (defined as <40 years at diagnosis) in the general population increased from 1.01% in 1997 (3) to 5.7% in 2010 (4). Patients with early-onset T2DM have been suggested to have similar or even worse metabolic profile than those with late-onset T2DM at the same ages and consequently (5, 6), higher risk of developing both macrovascular and microvascular complications (7–9). Our group reported that early-onset T2DM is at much increased risk of non-fatal cardiovascular disease (CVD) in China (10) but its effect size on microvascular disease in China remained largely uncertain.

Although the prevalence and incidence of diabetes are rather similar in the two sexes (4), gender-specific effects of diabetes on microvascular and macrovascular disease are likely. Female gender at reproductive age is a protective factor from coronary heart disease (CHD) in the non-diabetic population (11). However, in diabetic subjects, this observed protective effect is reversed (12, 13). In addition to sex hormones, women with diabetes were reported less likely to achieve the recommended targets of glycated hemoglobin (HbA1c), blood pressure (BP), and low-density lipoprotein cholesterol (LDL-C) as compared with their male counterparts (14, 15).

In non-diabetic animal models and human populations, male sex is characterized by a faster progression of renal impairment (16–18). Androgens are known to activate the renin–angiotensin system and might cause endothelial cell injury (19). Consistently, men with T2DM are at higher risk for microvascular complications such as severe retinopathy (20) and nephropathy (21) than women. However, it is uncertain whether early-onset T2DM has different effects on microvascular disease in Chinese men and women.

In this study, we further analyzed the data from the China National HbA1c Surveillance System (CNHSS) to address: (1) the effect size of early-onset T2DM on the life-time risk of microvascular disease, i.e., diabetic retinopathy (DR) and diabetic nephropathy (DN) in China; (2) gender differences in the risk of early-onset vs. late-onset T2DM for microvascular disease; and (3) whether the increased risk of microvascular disease in early-onset T2DM is attributable to longer durations of diabetes.

## Materials and Methods

### Research Design and Participants

A detailed description of the research design, study population, and methods was published previously (10). In brief, Chinese Diabetes Society launched an initiative, CNHSS in 2009 to monitor glycemic control of outpatients with T2DM in China in an effort to improve the quality of glycemic control. The current analysis used data collected from March 2012 to June 2012 in 630 hospitals, including 12 primary hospitals, 132 secondary hospitals, and 486 tertiary hospitals from 106 cities in 30 provincial administrative regions. During this period, research nurses or field workers invited the first seven patients who met the inclusion criteria in a consecutive way from patients seeking care at the outpatient clinics of department of endocrinology of the hospitals concerned as outpatients who met the inclusion and did not the following exclusion criteria in a consecutive way. The process continued until seven patients were successfully recruited per day with a total recruitment target of 400 patients for each hospital during the whole recruitment period unless the recruitment period ended. The inclusion and exclusion criteria were published elsewhere (10). Briefly, the inclusion criteria included being an outpatient with type 2 diabetes diagnosed by the 1999 World Health Organization’s criteria for diagnosis of diabetes and being treated with antidiabetic drugs; aged 18 years or older; having at least one previous outpatient medical record for diabetes; and being a local resident for at least 6 months consecutively before participation in the study. The exclusion criteria included the following: having type 1 diabetes, defined as acute presentation with diabetic ketoacidosis, heavy ketonuria, or continuous need for insulin within 1 year of diagnosis; having diabetes secondary to other diseases; being on diet and other lifestyle therapy or Chinese herbal medicine only; inpatients; pregnancy or breastfeeding; being unable to complete the survey owing to mental illness; being unconscious or unable to communicate. The study protocol was approved by the Ethics Committee of the Chinese People’s Liberation Army General Hospital, and written informed consent was obtained from each subject before collecting data.

### Data Collection Procedures

During the recruitment period, research nurses or postgraduate medical students who were familiar with the local area, had medical background knowledge were chosen as the fieldworkers. They reviewed the computerized medical notes of each recruited patient and recorded the data in a structured form. The collected information included gender, height, weight, BP, and date of diagnosis of diabetes. All laboratory evaluations were performed in the local hospitals where the interviews were conducted. Laboratory data on HbA1c and lipids were recorded. High density lipoprotein cholesterol (HDL-C) was calculated using the Friedewald’s equation (22). Other relevant data were obtained from face-to-face interview by the fieldworkers. Patients were required to report if they were diagnosed with any concomitant diseases or diabetes complications, including hypertension, CHD, dyslipidemia, cerebrovascular disease, DR, DN, diabetic neuropathy, diabetes-related foot ulcers, and others. They were also required to report information about the treatments used for the management of T2DM, including the use of oral antidiabetic drugs including dipeptidyl peptidase-4 inhibitors and glucagon-like peptide-1 receptor agonists, and different types of insulin, as well as combinations of these antidiabetes drugs. All the data collectors were trained on the basics of T2DM, the objectives of the study, the inclusion and exclusion criteria, data collection procedures, and details of the questionnaire. In addition, supervisors checked the reliability of the data collectors by re-interviewing a number of participants. All the data were inputted and uploaded to a central database by staff members.

### Definitions of Clinical Outcomes

Prior history of DN, DR, and other complications diagnosed by secondary or tertiary hospitals were retrieved from medical notes, including dates of diagnosis. DN was defined as having persistent proteinuria, i.e., urinary albumin excretion rate ≥20 µg/min or urinary albumin ≥30 mg/24 h after excluding other causes of kidney damage, urinary system infection, and blood in urine. The diagnosis of DR was made by ophthalmologist(s) based on typical changes of retinopathy on funduscopic examination due to diabetes, including background, pre-proliferative, proliferative, or maculopathy. Early-onset T2DM was defined as T2DM was diagnosed below 40 years of age.

### Statistical Analysis

Statistical analyses were performed using SAS version 9.1 (SAS Institute Inc., Cary, NC, USA) or SPSS 22 (SPSS Inc., Wacker Drive, Chicago, IL, USA). Student’s *t*-test or Kruskal–Wallis test was used to compare continuous variables where appropriate. We calculated age, gender, and levels of hospital specific prevalence of microvascular complications and further standardized the prevalence to Chinese population in 2011 (23), using the direct method.

We used binary logistic regression to estimate odds ratios (ORs) and 95% confidence intervals (CI) of early-onset vs. late-onset of T2DM for non-fatal microvascular disease. A structured adjustment scheme was used to control confounding effects of traditional risk factors with attention to whether these adjustments attenuated the effect sizes. Specifically, model 1 was adjusted for age and hospital levels; model 2 was adjusted for variables in model 1 and duration of diabetes; model 3 was adjusted for variables in model 2, metabolic profile and other traditional risk factors [body mass index (BMI), self-home glucose monitoring, diabetes medications as listed in Table 1, as well as levels of HbA1c, LDL-C, and SBP]; and model 4 was adjusted for targets of glycemic control (HbA1c < 7.0% or 53 mmol/mol) and lipid control (LDL-C < 2.6 mmol/L) in place of levels and other variables in model 3.

**Table 1:** Clinical and biochemical characteristics of patients by gender.

Variables	Male (*n* = 119,647)	Female (*n* = 102,890)	*P*
	Mean/number (SD or %)	Mean/number (SD or %)	
Age, years	58.1 ± 11.6	58.6 ± 11.1	<0.0001
Age of diabetes diagnosis	52.7 ± 11.1	52.9 ± 10.6	<0.0001
Early-onset of diabetes	15,622 (13.1%)	11,341 (11.0%)	<0.0001
Duration of diabetes, years	5.4 ± 4.9	5.7 ± 5.3	<0.0001
BMI, kg/m^2^	24.6 ± 2.8	24.4 ± 3.3	<0.0001
BMI groups			<0.0001
≤23.9	50,567 (42.3%)	48,026 (46.7%)	
24.0–27.9	57,134 (47.8%)	43,033 (41.8%)	
≥28.0	11,946 (10.0%)	11,831 (11.5%)	
HbA1c, %	7.8 ± 1.6	7.7 ± 1.6	<0.0001
HbA1c, mmol/mol	61.3 ± 17.3	60.8 ± 17.0	<0.0001
The targets of glycemic control achieved	35,027 (29.3%)	31,902 (31.0%)	<0.0001
SBP, mmHg	131.9 ± 14.3	131.2 ± 14.8	<0.0001
DBP, mmHg	82.2 ± 10.3	80.7 ± 10.1	<0.0001
The targets of BP control achieved	70,230 (58.7%)	64,102 (62.3%)	<0.0001
Triglyceride, mmol/L	1.8 (1.3–2.8)	1.7 (1.3–2.6)	<0.0001
LDL-C, mmol/L	3.0 ± 1.6	3.1 ± 1.6	0.0326
HDL-C, mmol/L	1.3 ± 0.9	1.4 ± 0.9	<0.0001
The targets of lipid control achieved	23,814 (19.9%)	11,472 (11.2%)	<0.0001
Diabetes medications			
One OAD only	23,217 (19.4%)	21,552 (20.9%)	<0.0001
Two OADs only	30,992 (25.9%)	27,732 (26.9%)	<0.0001
Three OADs only	7,958 (6.7%)	6,949 (6.8%)	0.3345
Four and more OADs	316 (0.3%)	314 (0.3%)	0.0691
OADs plus insulin	28,848 (24.1%)	23,937 (23.3%)	<0.0001
OADs plus GLP-1	265 (0.2%)	157 (0.2%)	0.0002
Insulin only	27,824 (23.3%)	22,098 (21.5%)	<0.0001
Hospital levels			<0.0001
Primary	2,203 (1.8%)	2,075 (2.0%)	
Secondary	23,965 (20.0%)	22,289 (21.6%)	
Tertiary	93,479 (78.1%)	78,526 (76.3%)	
Non-fatal macrovascular complications			
Non-fatal CHD	12,683 (10.6%)	11,612 (11.3%)	<0.0001
Non-fatal stroke	4,898 (4.1%)	4,070 (4.0%)	0.0988
Non-fatal CVD	15,931 (13.3%)	14,149 (13.7%)	0.0027
Non-fatal microvascular complications			
Diabetic nephropathy	6,814 (5.7%)	5,244 (5.1%)	<0.0001
Diabetic retinopathy	9,857 (8.2%)	9,071 (8.8%)	<0.0001
Either nephropathy or retinopathy	14,293 (12.0%)	12,394 (12.1%)	0.4695

*BMI, body mass index; HbA1c, glycated hemoglobin; SBP/DBP, systolic/diastolic blood pressure; LDL-C, low-density lipoprotein cholesterol; HDL-C, high density lipoprotein cholesterol; CHD, coronary heart disease; CVD, cardiovascular disease defined as having either CHD or stroke; BP, blood pressure*.

*The targets of glycemic control were defined as HbA1c < 7% no matter they were on oral antidiabetic drugs or insulin; the targets of BP control were defined as SBP/DBP < 140/90 mmHg no matter whether they were on antihypertensive drugs; the targets of lipid control were defined as LDL-C < 2.6 mmol/L, HDL-C > 1.0 mmol/L in men and >1.3 mmol/L in women, and triglyceride <1.7 mmol/L whether they were on lipid-lowering drugs or not; and macrovascular or microvascular complications of patients were based on medical record*.

*Data are mean (SD), median (IQR), or *n* (%)*.

*P values were derived from chi-square test or Student’s t-test*.

To investigate additive interaction between gender and early-onset T2DM for the risk of microvascular complications, we obtained three indicators of additive interaction using a calculator provided by Andersson et al.: relative excess risk due to interaction (RERI), attributable proportion due to interaction (AP) and synergy index (S) with 95% CI using the delta method (24). RERI > 0, AP > 0, or S > 1 indicates a significant additive interaction or significant enhancing or alleviating effect. To observe the age at which the risk of non-fatal microvasular diseases started to rise, we transformed the cross-sectional survey into a retrospective cohort with follow-up time calculated as time in years from birth to the date of occurrence of non-fatal microvasular disease or the date of the survey, whichever came first. Kaplan–Meier analysis was used to observe the risk of microvascular disease from birth to the date of the survey. All *P* values were two-tailed and the level of significance was set at 0.05.

## Results

### Clinical Characteristics of Study Patients

Among 223,612 enrolled patients, 1,075 with missing key variables were excluded, and the remaining 222,537 were used in the analysis, with a mean age of 58.3 years (SD: 11.4) and a mean duration of diabetes of 5.6 years (SD 5.1). Among these patients, 4,278 (1.9%) were recruited from primary care hospitals, 46,254 (20.8%) from secondary care hospitals, and 172,005 (77.3%) from tertiary care hospitals. Male patients account for 53.8%. Clinical characteristics of these patients by gender were presented in Table 1. Female patients were older, had an older age at being diagnosed with T2DM and a longer duration of diabetes than male patients. The levels of BMI, HbA1c, SBP, DBP, and triglyceride were higher, but the levels of LDL-C and HDL-C were lower in male patients than in female patients. Male patients were more likely to achieve the lipid control target but less likely to achieve the glycemic control and BP control targets. Also, male patients were more likely to use insulin alone or insulin combined with oral antidiabetic drugs.

### Prevalence of Non-Fatal Microvascular Diseases by Gender

The age-standardized prevalence of non-fatal DN, DR, and microvascular diseases was significantly higher in male patients than in female patients (male vs. female: 4.5 vs. 3.0% for DN, 5.3 vs. 5.1% for DR, and 8.4 vs. 7.1% for either of them). Furthermore, male patients had a significantly higher prevalence of non-fatal DN than female patients in each of the age groups younger than 65 years and the risk reversed in the groups aged 65 years and older, but without significant differences. The prevalence of non-fatal DR in male was higher than in female in each of the age groups younger than 55 years, with *P* < 0.05 except for groups aged <35 and 50–54 years, but the risk reversed in the groups of 55 years and older, with *P* < 0.05 except for the group aged 60–64 years (Table 2).

**Table 2:** Age-standardized prevalence of non-fatal microvascular diseases among patients with type 2 diabetes by gender.

Age group, years	Prevalence of nephropathy			Prevalence of retinopathy			Prevalence of either		
	Male	Female		Male	Female		Male	Female	
		
	*n*(%)	*n*(%)	*P*	*n*(%)	*n*(%)	*P*	*n*(%)	*n*(%)	*P*
**Standardized rate by age among the whole study patients**									
<35	103 (3.8)	32 (1.6)	0.0001	89 (3.3)	52 (2.7)	0.2506	165 (6.0)	75 (3.9)	0.0009
35–39	160 (4.0)	57 (2.0)	0.0001	169 (4.2)	80 (2.8)	0.0020	285 (7.1)	123 (4.3)	<0.0001
40–44	332 (4.5)	157 (3.0)	0.0001	455 (6.1)	262 (5.1)	0.0133	687 (9.2)	349 (6.8)	<0.0001
45–49	675 (4.7)	356 (3.2)	0.0001	937 (6.5)	606 (5.4)	0.0004	1,356 (9.4)	847 (7.6)	<0.0001
50–54	720 (4.7)	529 (3.8)	0.0003	1,015 (6.6)	901 (6.5)	0.7932	1504 (9.8)	1,251 (9.1)	0.0353
55–59	1,004 (4.7)	737 (3.7)	<0.0001	1,579 (7.4)	1,593 (8.0)	0.0181	2,231 (10.5)	2,052 (10.3)	0.6830
60–64	1,068 (6.0)	897 (5.4)	0.0076	1,604 (9.1)	1,617 (9.7)	0.0521	2,287 (12.9)	2,181 (13.0)	0.7301
65–69	876 (5.5)	830 (5.9)	0.1357	1460 (9.1)	1,481 (10.5)	<0.0001	2,046 (12.8)	2,010 (14.2)	0.0003
≥70	1,876 (9.1)	1,649 (9.6)	0.1210	2549 (12.4)	2,479 (14.4)	<0.0001	3,732 (18.1)	3,506 (20.3)	<0.0001
Total	6,814 (5.7)	5,244 (5.1)	<0.0001	9,857 (8.2)	9,071 (8.8)	<0.0001	14,293 (12.0)	12,394 (12.1)	0.4695
*P*^a^	<0.0001	<0.0001		<0.0001	<0.0001		<0.0001	<0.0001	
Standardized^b^ (%)	4.5	3.0		5.3	5.1		8.4	7.1	

**Standardized rate by different levels of hospitals (%)**									
Primary hospitals	4.2	1.8		2.0	3.2		6.1	4.5	
Secondary hospitals	4.8	2.9		4.6	4.8		8.2	6.6	
Tertiary hospitals	4.5	3.1		5.5	5.1		8.6	7.2	

*^a^P for trend*.

*^b^The prevalence was standardized to the population of mainland China in 2011*.

### Risk of Early-Onset vs. Late-Onset of T2DM for Microvascular Disease by Gender

After adjustment for age and levels of hospitals, early-onset T2DM was associated with increased risk of non-fatal DN, non-fatal DR, and either of them in men and women. However, the effect sizes were higher in men than in women (but not for non-fatal DR), with a 3.30 (95% CI: 3.03–3.60) fold risk for DN in men and a 3.19 (95% CI: 2.87–3.55) fold risk in women, a 2.67 (95% CI: 2.51–2.85) fold risk for either of them in men and a 2.53 (95% CI: 2.35–2.72) fold risk in women. Additional adjustment for duration of diabetes and other factors attenuated the effect sizes, but men remained at higher risks of DN, DR, and either of them than women. All the associations remained significant in men but not in women (Table 3).

**Table 3:** Odds ratio (OR) of early-onset vs. late-onset of type 2 diabetes for non-fatal microvascular disease by gender.

	Nephropathy		Retinopathy		Either of them	
	Male	Female	Male	Female	Male	Female
		
	OR (95% CI)	OR (95% CI)	OR (95% CI)	OR (95% CI)	OR (95% CI)	OR (95% CI)
**Multivariable model 1**						
Early- vs. late-onset	3.30 (3.03–3.60)	3.19 (2.87–3.55)	2.47 (2.29–2.66)	2.50 (2.29–2.71)	2.67 (2.51–2.85)	2.53 (2.35–2.72)
**Multivariable model 2**						
Early- vs. late-onset	1.40 (1.27–1.53)	1.24 (1.10–1.39)	1.24 (1.14–1.34)	1.09 (0.99–1.19)	1.31 (1.22–1.40)	1.10 (1.01–1.19)
**Multivariable model 3**						
Early- vs. late-onset	1.34 (1.22–1.47)	1.20 (1.07–1.35)	1.18 (1.09–1.28)	1.04 (0.95–1.14)	1.25 (1.17–1.34)	1.05 (0.97–1.14)
**Multivariable model 4**						
Early- vs. late-onset	1.37 (1.24–1.50)	1.22 (1.09–1.37)	1.21 (1.11–1.31)	1.06 (0.97–1.17)	1.28 (1.19–1.37)	1.07 (0.99–1.16)

*BMI, body mass index; SHGM, self-home glucose monitoring; HbA1c, glycated hemoglobin; LDL-C, low-density lipoprotein cholesterol; CI, confidence intervals; BP, blood pressure*.

*Multivariable model 1 was adjusted for age and hospital levels*.

*Multivariable model 2 was adjusted for age, hospital levels, and duration of diabetes*.

*Multivariable model 3 was adjusted for age, hospital levels, duration of diabetes, BMI, SHGM, diabetes medications (as listed in Table 1), as well as HbA1c, LDL-C, and SBP (227 men and 151 women were excluded due to missing values)*.

*Multivariable model 4 was adjusted for age, hospital levels, duration of diabetes, BMI, SHGM, diabetes medications (as listed in Table 1), as well as the targets of glycemic, BP, and LDL-C control (227 men and 151 women were excluded due to missing values)*.

### Synergistic Effect of Male Gender and Early-Onset T2DM

**Figure 1:**
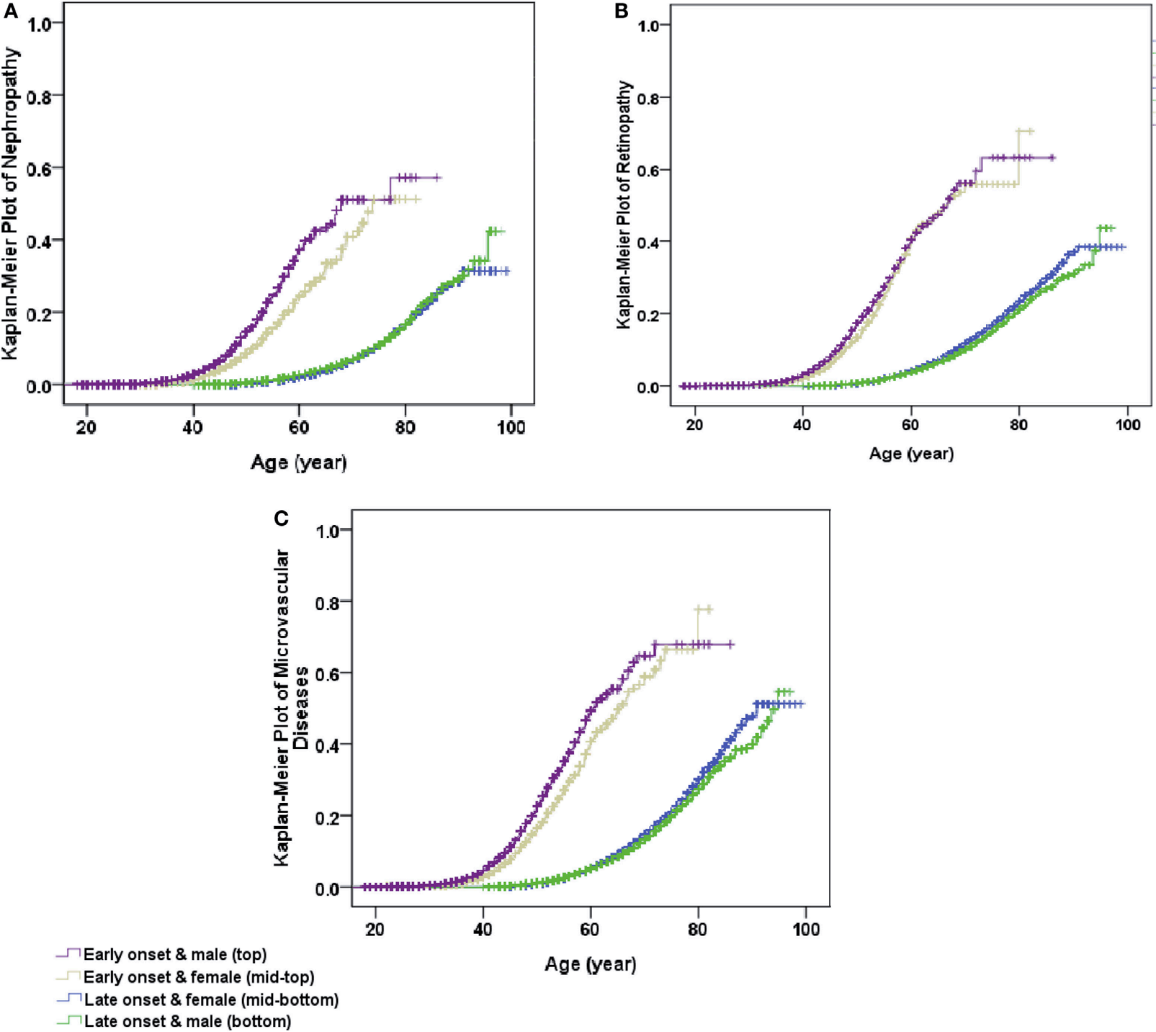
Kaplan–Meier plot of non-fatal microvascular diseases stratified by early- and late-onset of diabetes by gender. **(A)** Non-fatal nephropathy, **(B)** non-fatal retinopathy, and **(C)** non-fatal microvascular diseases (either non-fatal nephropathy or non-fatal retinopathy). All *P* values for Log rank test <0.001.

After adjustment for age and levels of hospitals in model 1, using female patients with late-onset T2DM as the reference, the co-presence of early-onset T2DM and male gender significantly enhanced the effect sizes of presence of either early-onset alone (OR: 2.30, 95% CI: 2.15–2.46) or male gender alone (OR: 0.96, 95% CI: 0.93–0.99) to 2.79 (95% CI: 2.63–2.96) for microvascular disease, with significant interactions (RERI: 0.54, 95% CI: 0.35–0.73; AP: 0.19, 95% CI: 0.13–0.25; and SI: 1.43, 95% CI: 1.25–1.63). Additional adjustment for duration of diabetes in model 2 attenuated the effect sizes, but the presence of both early-onset and male gender remained to significantly increase the risk of DN (RERI: 0.53, 95% CI: 0.37–0.68; AP: 0.30, 95% CI: 0.23–0.38; and SI: 3.32, 95% CI: 1.97–5.60), DR (RERI: 0.14, 95% CI: 0.03–0.25; AP: 0.11, 95% CI: 0.03–0.20; and SI: 2.19, 95% CI: 0.96–4.99), and either of them (RERI: 0.31, 95% CI: 0.21–0.41; AP: 0.22, 95% CI: 0.15–0.28; and SI: 3.36, 95% CI: 1.77–6.39) than presence of either early-onset alone or male gender alone, with significant additive interaction. In addition, the results were similar when further adjustment for other confounding factors in model 3 and model 4 (Table 4). Kaplan–Meier plots showed that the cumulative incidence of DN and microvascular disease started to rise at 40 years of age in men with early-onset T2DM, and at 45 years of age in women with early-onset T2DM, much earlier than 60 years of age in both men and women with late-onset T2DM. On the other hand, the cumulative incidence of DR started to rise at 40 years of age in both men and women with early-onset T2DM, much earlier than 60 years of age in both men and women with late-onset T2DM (Figure 1).

**Table 4:** Interaction between gender and early-onset type 2 diabetes mellitus for non-fatal microvascular disease.

	Nephropathy	Retinopathy	Either
	OR (95% CI)	OR (95% CI)	OR (95% CI)
**Model 1**			
Late-onset and female	1.00	1.00	1.00
Late-onset and male	1.08 (1.03–1.12)	0.91 (0.88–0.94)	0.96 (0.93–0.99)
Early-onset and female	2.79 (2.54–3.08)	2.32 (2.15–2.50)	2.30 (2.15–2.46)
Early-onset and male	3.96 (3.65–4.29)	2.40 (2.24–2.58)	2.79 (2.63–2.96)
RERI	1.09 (0.75–1.43)	0.18 (−0.03 to 0.38)	0.54 (0.35–0.73)
AP	0.28 (0.20–0.35)	0.07 (−0.01 to 0.16)	0.19 (0.13–0.25)
SI	1.58 (1.36–1.84)	1.14 (0.98–1.34)	1.43 (1.25–1.63)
**Model 2**			
Late-onset and female	1.00	1.00	1.00
Late-onset and male	1.14 (1.09–1.19)	0.94 (0.91–0.98)	1.00 (0.97–1.03)
Early-onset and female	1.09 (0.98–1.21)	1.18 (1.09–1.27)	1.14 (1.06–1.22)
Early-onset and male	1.76 (1.61–1.91)	1.26 (1.17–1.36)	1.45 (1.36–1.54)
RERI	0.53 (0.37–0.68)	0.14 (0.03–0.25)	0.31 (0.21–0.41)
AP	0.30 (0.23–0.38)	0.11 (0.03–0.20)	0.22 (0.15–0.28)
SI	3.32 (1.97–5.60)	2.19 (0.96–4.99)	3.36 (1.77–6.39)
**Model 3**			
Late-onset and female	1.00	1.00	1.00
Late-onset and male	1.12 (1.08–1.17)	0.93 (0.90–0.96)	0.98 (0.95–1.01)
Early-onset and female	1.08 (0.97–1.19)	1.13 (1.04–1.22)	1.09 (1.03–1.17)
Early-onset and male	1.63 (1.50–1.78)	1.15 (1.07–1.23)	1.31 (1.23–1.40)
RERI	0.44 (0.29–0.59)	0.09 (−0.02 to 0.20)	0.25 (0.15–0.34)
AP	0.27 (0.19–0.35)	0.08 (−0.01 to 0.17)	0.19 (0.12–0.26)
SI	3.23 (1.76–5.91)	2.67 (0.46–15.23)	4.60 (1.33–15.91)
**Model 4**			
Late-onset and female	1.00	1.00	1.00
Late-onset and male	1.10 (1.05–1.14)	0.91 (0.88–0.94)	0.96 (0.93–0.99)
Early-onset and female	1.09 (0.98–1.21)	1.14 (1.05–1.24)	1.10 (1.03–1.19)
Early-onset and male	1.63 (1.50–1.78)	1.16 (1.08–1.25)	1.32 (1.24–1.41)
RERI	0.45 (0.30–0.60)	0.11 (0.01–0.22)	0.27 (0.17–0.36)
AP	0.28 (0.20–0.36)	0.09 (0.01–0.18)	0.20 (0.13–0.27)
SI	3.48 (1.81–6.70)	3.09 (0.50–19.23)	5.45 (1.31–22.78)

*BMI, body mass index; SHGM, self-home glucose monitoring; HbA1c, glycated hemoglobin; LDL-C, low-density lipoprotein cholesterol; RERI, relative excess risk due to interaction; AP, attributable proportion due to interaction; SI, synergy index; CI, confidence intervals; BP, blood pressure; OR, odds ratio*.

*Multivariable model 1 was adjusted for age and hospital levels*.

*Multivariable model 2 was adjusted for age, hospital levels, and duration of diabetes*.

*Multivariable model 3 was adjusted for age, hospital levels, duration of diabetes, and BMI*.

*SHGM, diabetes medications (as listed in Table 1), as well as HbA1c, LDL-C, and SBP (227 men and 151 women were excluded due to missing values)*.

*Multivariable model 4 was adjusted for age, hospital levels, duration of diabetes, BMI, SHGM, diabetes medications (as listed in Table 1), as well as the targets of glycemic, BP, and LDL-C control (227 men and 151 women were excluded due to missing values)*.

## Discussion

Diabetes imparted a larger risk on macrovascular disease to women than to men, including CHD and stroke (12, 13). However, there are fewer studies that examined gender differences in microvascular diseases in T2DM. A community-based cross-sectional study showed that female patients with T2DM had a higher prevalence of proteinuria and renal impairment than males with an increasing prevalence by age (25). In the United Kingdom Prospective Diabetes Study, DR was more prevalent in men than in women, and this could not be explained by the major risk factors, since women had higher BP and higher glucose levels than men (26). A cohort study with 1,146 participants with T2DM found that men showed a faster progression rate of renal function (18). Consistently, our study found that the age-standardized prevalence of non-fatal DN, DR, and either of them was significantly higher in men than in women with T2DM.

Although most studies indicated that women with T2DM were less likely to achieve HbA1c, BP, and LDL-C targets than men with T2DM (14, 15), men were still at higher risk for microvascular complications than women (18, 26). Sex hormones were considered to play a role in these gender differences in microvascular disease (25). A double-blind, placebo-controlled clinical trial reported that estrogen and estrogen plus medroxyprogesterone acetate produced a significant reduction in LDL-C levels and a significant increase in HDL cholesterol levels (27). Furthermore, several studies also found that short-term administration of estrogens in recombination with a synthetic progestin reduced proteinuria and improved creatinine clearance in postmenopausal women with T2DM (28, 29). On the other hand, androgens are known to activate the renin–angiotensin system and might cause endothelial cell injury (19). Given that estrogen levels decline with increasing age, it is plausible that high estrogens before menopause may play a protective role from microvascular disease, too. Consistently, our analysis observed that in early-onset T2DM, DN developed 5 years earlier in men than in women. This gender-specific effect can be partially explained by higher BMI and worsened metabolic profile such as higher HbA1c and higher BP in men than in women. Nevertheless, the residual risk remained to be significant and substantial, suggesting that changes in protective effects of estrogen or harmful effects of androgens (29) in the long course of T2DM remain to play an important role in the risk of microvascular disease in T2DM.

Our findings have important clinical implications. It is estimated that China had more than 5.7 million people with early-onset diabetes (4). These people were at high life-time risk of CVD (10). This analysis provided further evidence that DN developed 20 years earlier in men and 15 years earlier in women, and DR developed 20 years earlier in both genders, in early-onset T2DM than in late-onset T2DM. Our study highlights the importance of prevention of T2DM in people before 40 years of age. Once T2DM developed in the young group, good metabolic control of hyperglycemia, hypertension, and abnormal lipids is essential to reduce the burden of macrovascular and microvascular disease (30, 31).

This study had limitations. First, DN and DR were ascertained by reviewing medical records, not by universal screening. Some medical records were not available. As a result, the true prevalence of microvascular complications might be under estimated. Second, the inclusion and exclusion criteria only recruited patients on hypoglycemic drug treatments. Patients on diet management only were missed. Also, data for lifestyle factors such as smoking status, alcohol consumption, dietary intake, and exercise were also not collected. However, in our previous study (10), the validation results showed that exclusion of patients with diet only and non-adjustment for lipid-lowering and antihypertensive drugs resulted in marginal changes in ORs for risk of non-fatal CVD in patients with early-onset vs. late-onset T2DM. Third, this was a cross-sectional study, so competing risk of death could not be considered. Since long duration of diabetes was associated with increased risk of death (32), more deaths were expected in patients with early-onset T2DM than in those with late-onset T2DM at the same ages, which might lead to underestimation of the risk. Fourth, we have used computerized medical records in data retrieval. Although it increased the speed of data retrieval, variations in the electronic medical record formats made data sharing and comparability across systems a challenge. However, most of the data we have retrieved from computerized medical records are demographic information, so it has a relatively limited impact. Fifth, diagnostic bias may exist due to the availability of infrastructures at different hospitals. To minimize the possible bias, all doctors in the 630 hospitals were required to use the same definitions when the diagnoses were established. In addition, only diagnosis of DN, DR, and other complications by secondary or tertiary hospitals were recorded.

In summary, early-onset T2DM imparted higher risk of microvascular complications to Chinese men than to Chinese women. Our study suggests that more medical resources should be shifted to prevention of T2DM in the young people at risk and shifted to tight control of hyperglycemia and other metabolic risk factors in early-onset T2DM, especially among Chinese men, so to reduce the burden of microvascular complications in T2DM in China.

## Ethics Statement

The study protocol was approved by the Ethics Committee of the Chinese People’s Liberation Army General Hospital and informed consent was obtained from each subject before collecting data.

## Author Contributions

XY, LJI, XH, and JZ conceived the idea. LJI, JLU, and XG designed the CNHSS and collected the data for the CNHSS. XH, JZ, and XY analyzed the data and wrote the first draft. All the authors gave critical comments, contributed to the writing of the manuscript, and agreed to submit and publish the manuscript. XY and LJI (the corresponding authors) and XH and JZ (the first authors) take full responsibility for the work as a whole, including the study design, access to the data, and decision to submit.

## Conflict of Interest Statement

The authors declare that the research was conducted in the absence of any commercial or financial relationships that could be construed as a potential conflict of interest.
